# Activation tagging in *Salvia miltiorrhiza* can cause increased leaf size and accumulation of tanshinone I and IIA in its roots

**DOI:** 10.1186/1999-3110-54-37

**Published:** 2013-09-24

**Authors:** Hsin-Shueh Ho, Rishi Kishore Vishwakarma, Emily Chin-Fun Chen, Hsin-Sheng Tsay

**Affiliations:** 1 Department of Applied Chemistry, Chaoyang University of Technology, 168, Jifong E Road, Taichung, Wufong, 41349 Taiwan; 2 Department of Agronomy, National Chung Hsing University, Taichung, Taiwan

**Keywords:** Activation-tagging, HPLC, Tanshinones, *Salvia miltiorrhiza*, Scanning electron microscopy

## Abstract

**Background:**

*Salvia miltiorrhiza* Bunge (Danshen), an important herb in traditional Chinese medicine, is commonly used for treatment of cardiovascular diseases. One of the major bioactive constituents of Danshen, diterpenoid tanshinone, has been proved with pharmacological properties and have the potential to be a new drug candidate against various diseases. In our previous study, we have established an activation tagging mutagenesis (ATM) population of callus lines of *S*. *miltiorrhiza* Bunge by *Agrobacterium*- mediated transformation.

**Results:**

In the present study, we have identified ATM transgenic *Salvia* plant (SH41) with different leaf morphology and more tanshinones in its roots. The transgenic background of SH41 was identified by PCR (using *hpt* II primers) and Southern blots. PCR analysis showed a single band of *hpt* II gene and Southern blot analysis showed single insertion in SH41. External appearance of ATM transgenic SH41 was observed with broader leaves comparing to non-transformed plants. More healthy trichomes as well as bigger and wobbly guard cells and stomata were observed in SH41 by scanning electron microscopy (SEM). Quantitative analysis of active compounds in SH41 roots revealed a significant increase in tanshinone I (3.7 fold) and tanshinone IIA (2 fold) contents as compared to the wild plant.

**Conclusions:**

We have generated an activation tagged transgenic *Salvia* plant (SH41) with different leaf morphology and high diterpenes content in its roots. The increased amount of tanshinones in SH41 will definitely offer a route for maximizing the benefits of this plant in traditional Chinese herbal medicines. The present report may also facilitate the application of ATM for genetic manipulation of other medicinal crops and subsequent improved metabolite contents.

**Electronic supplementary material:**

The online version of this article (doi:10.1186/1999-3110-54-37) contains supplementary material, which is available to authorized users.

## Background

*Salvia miltiorrhiza* Bunge (Danshen) is a recognized traditional Chinese medicine and is broadly used for the treatment of various cardiovascular and cerebrovascular diseases. This wide range of medicinal uses is mainly due to its property of stimulating blood circulation and removing blood stasis (Zhou et al. 2012). Danshen contained water soluble phenolics including danshensu, protocatechuic aldehyde, protocatechuic acid, caffeic acid, rosmarinic acid and salvianolic acid A & B as well as lipid soluble diterpene quinones: cryptotranshinone, transhinone I and transhinone IIA (Liu et al. 2007). Phenolics and quinones have alike individual pharmacological properties such as antioxidant, anti-apoptosis and vasodilation (Zhou et al. 2005; Lam et al. 2007; Lam et al. 2008a; Wang et al. 2011a).

Many experimental and clinical investigations have reported that tanshinones and their derivatives can prevent or slow the development of a various diseases including hypoxia/reoxygenation injury, cardiovascular diseases, cancer, neonatal hypoxic ischemic encephalopathy, hepatic fibrosis as well as neuro degenerative diseases (Wu et al. 1993; Yagi et al. 1994; Yoon et al. 1999; Takahashi et al. 2002; Han et al. 2008; Shu et al. 2010).

Due to its extensive medicinal values, *S*. *miltiorrhiza* has been studied by various research groups over the world, especially in biotechnological and *in vitro* propagation field. Through several strategies like genetic manipulation of biosynthetic pathway via genetic transformation (Chen et al. 1997; Lee et al. 2008; Kai et al. 2011), hairy root cultures (Hu et al. 1993), use of plant growth regulators and elicitors (Wu et al. 2003; Zhang et al., 2004; Ge and Wu 2005,2005; Gupta et al. 2011), success has been reported in enhancing the production of secondary metabolites in *S. miltiorrhiza*.

Although, several reports represented the micropropagation and genetic transformation methods for *S. miltiorrhiza* by other means (Zhang et al. 1995; Chen et al. 1997; Zhang et al. 1997; Yan and Wang 2007; Lee et al. 2008), but there is a limited report available on activation tagging mutagenesis. Therefore, the overall objective of the current research was to develop and investigate the effect of ATM on morphological as well as histological changes and tanshinones content in transgenic *Salvia* plant.

## Methods

### Plant material

*S*. *miltiorrhiza* used for this study was collected from Zhengzhou City, Henan Province Jiyuan County mountains, China. Activation tagged mutagenic (ATM) *Salvia* plants are maintained at greenhouse in Chaoyang University of Technology.

### Gene constructs and bacterial strain

*A. tumefaciens* strain EHA105 harboring a binary vector pTAG8 (Hsing et al. 2007; Chen et al. 2009; Tsay et al. 2012) was used for the genetic transformation of *S. miltiorrhiza*. The T-DNA region of the binary vector contained a selectable marker coding for the hygromycin phosphotransferase (*hpt* II) gene under control of CaMV 35S promoter (provided by Dr. Su-May Yu, Academia Sinica, Taipei, Taiwan).

### Genetic transformation and confirmation of ATM insertion in *Salvia* plant

*Salvia* transgenic raised by Lee et al. (2008) was used for this study. ATM transgenic *S. miltiorrhiza* plant (SH41) with an altered morphological appearance was investigated. The transformation was confirmed by PCR detection of hygromycin phsphotransferase gene (*hpt* II) gene using genomic DNA and gene specific primers *hpt* II F 5′-GTCGTGGCGATCCTGCAAGC-3′ and *hpt* II R 5′-CCTGCGGGTAAATAGCTGCGC-3′. Total genomic DNA was isolated from young leaves of SH41 and control plants using ZR plant/seed DNA MiniPrep kit (Zymo Research). The PCR reaction contained 50 ng genomic DNA, 0.2 mM dNTPs mix, 500 nM each forward and reverse primers, and 1U of Taq DNA polymerase. The PCR was carried out in a thermal cycler (BIO-RAD, USA) under the following conditions: 1 cycle of 94°C (5 min), 35 cycles of 94°C (30 s), 55°C (30 s) and 72°C (1 min), and final extension at 72°C (7 min). The plasmid pTAG8 was used as a positive control. The amplification products were analyzed on 1% agarose gel by electrophoresis and visualized under UV light after ethidium bromide staining.

The insertion of the gene was further confirmed by Southern blot analysis. Genomic DNA of SH41 and control plants was isolated using the modified cetyl trimethyl ammonium bromide (CTAB) method (Doyle and Doyle 1987). Isolated genomic DNA (15 μg) was digested overnight with restriction enzymes *Pst* I, *Hind* III and *Eco* RI in appropriate buffer at 37°C. The digested DNA was electrophoresed on 0.8% agarose gel in 1X Tris-acetate/EDTA buffer. The gel was then treated with dupurination, denaturation and neutralization buffers and DNA was blotted onto a positively charged nylon membrane (Bio-Rad) as described by Sambrook et al. (1989). A PCR-amplified *hpt* II gene fragment was labelled with digoxigenin-9-dUTP using Dig High Prime Labeling and Detection Starter Kit I (Roche, USA) and used as a probe. After prehybridization for 2 h at 52°C, labeled probe was added and kept for hybridization at the same temperature for 16 h in hybridization chamber. The membrane was subjected to a series of stringency washes (high stringency at 56°C) to eliminate nonspecific probe binding. The signals were immuno-detected with anti-digoxigenin-AP and visualized with the colorimetric substrates BCIP/NBT according to manufacturer’s instruction.

### Scanning electron microscopy (SEM) analysis

In order to investigate the differences observed in the morphology of the wild type (WT) and transgenic (SH41) *Salvia* plants, scanning electron microscopy (SEM) was performed. *Saliva* leaf and root samples were cut into an appropriate size, adhered to the metal base, frozen in liquid nitrogen and scanned in low-vacuum SEM (JEOL JSM-633OF) with chamber pressure of 30 Pa and an accelerated voltage 15 kV.

### Quantitative (HPLC) analysis

High performance liquid chromatography (HPLC) was performed to detect the water soluble metabolites danshensu, protocatechuic acid, protocatechuic aldehyde, caffeic acid, rosmarinic acid, salvianolic acid A and salvinic acid B, as well as lipid soluble tanshinone I, tanshinone ΠA and cryptotanshinone in roots. All the standards were obtained from Sigma and Formosa Kingstone Bioproducts International Corp.

First, extraction was optimized using different solvent system with commercially available *Salvia* plant roots (CK; purchased from Joint Pharmacy, Taichung City, Taiwan). For further analysis, 50 mg dried roots of 4 months old transgenic (SH41) and wild type *Salvia* plants was finely ground by mortar pestle using liquid nitrogen and extracted with 10 ml of 60% ethanol containing 0.05% formic acid by sonication for 10 min along with and commercially available (CK) *Salvia* root powder. The extracts were filtered through filter paper and vacuum dried. Further, the dried samples were dissolved in methanol/water (1:1), filtered through 0.22 μm membrane and subjected to HPLC analysis. The HPLC system consisted of a pump (Hitachi L-2130), automatic injector (Hitachi L-2200) and Diode Array Detector (Hitachi L-2450). The column used was C18 (Waters; 5 um, 4.6 × 250 mm) and UV detection was carried out at 280 nm. The mobile phase acetonitrile/water containing 0.05% H_3_PO_4_ was pumped at a flow rate was 1.0 mL/min and programmed as follows: 10% acetonitrile for 0 min; 40% acetonitrile for 15 min; 60% acetonitrile for 15 min and 75% acetonitrile for 30 min. Dilutions (1.512, 3.125, 6.25, 12.5, 25, 50, 100 mg/mL) of standard compounds were prepared and 10 μL aliquots were subjected to HPLC. This was repeated thrice and calibration plots were constructed from the peak areas of standard compounds.

### Statistical analysis

Each result shown in tables was the mean of at least three replicated experiments and the standard deviation (±SD) value was calculated.

## Results

### Morphological investigation of activation tagged *Salvia* plant

**Figure 1:**
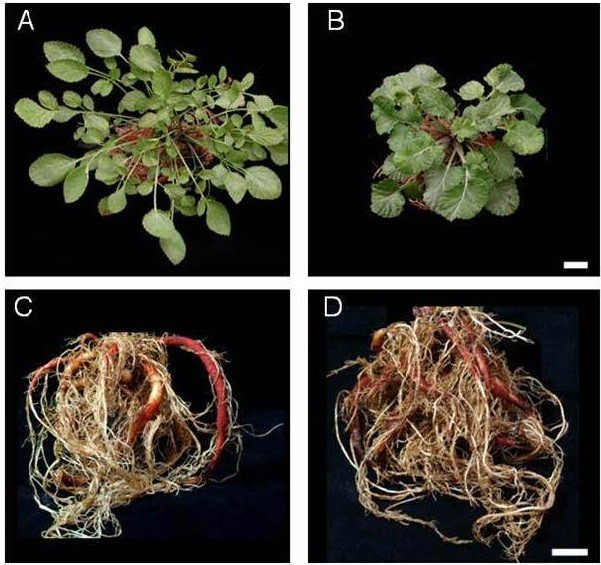
***S*** **.** ***miltiorrhiza*** **plant morphology.** **(A)** Wild type (WT)- above ground. **(B)** SH41- above ground. **(C)** WT- roots. **(D)** SH41- roots. Scale bar = 1 cm.

As shown in Figure 1, we can clearly see the morphological difference between SH41 and wild type plant. SH41 plant exhibited larger sized leaves as well as thick and smaller petiole than wild type (Figure 1).

**Figure 2:**
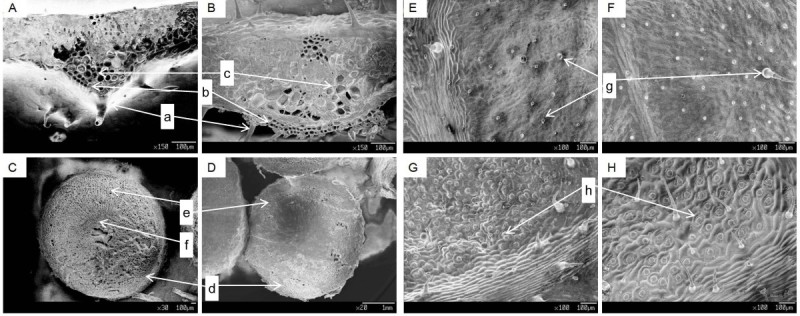
**SEM images showing morphological differences. (A & B)** Leaf blade cross-sections of WT and SH41 respectively. **(C & D)** Root cross sections of WT and SH41 respectively. **(E & F)** Adaxial leaf surface of WT and SH41 respectively. **(G & H)** Abaxial leaf surface of WT and SH41 respectively. (a & g) Trichomes, (b) Palisade tissue, (c) Spongy tissue, (d) Cork layer, (e) Cortex, (f) Formation of the layer, and (h) Guard cells.

**Figure 3:**
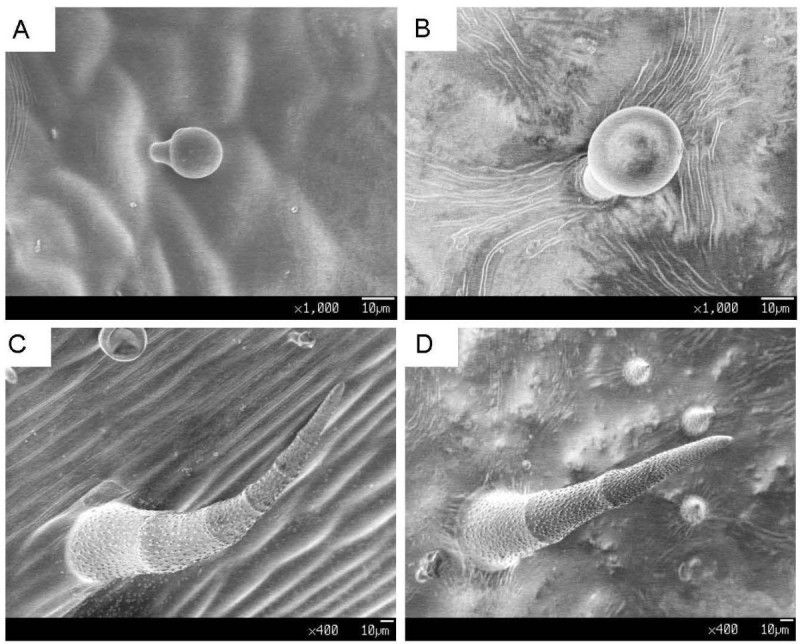
**SEM image showing two forms of hairy structure (trichomes).** **(A & B)** Flagpole-shaped trichome of WT and SH41 respectively, and **(C & D)** Bamboo shoot shaped trichome of WT and SH41 respectively.

The microscopic (SEM) analysis showed that activation tag mutagenesis affected the morphological development of leaf tissues during growth. Cross section of leaf blades showed thickening of the palisade and the spongy tissues were slightly larger in SH41 as compared to wild type (Figures 2A, 2B). No significant differences were observed in the morphology of roots (Figures 2C, 2D). Adaxial and abaxial surface of SH41 leaf showed more trichomes than WT with bigger and wobbly guard cells and stomata (Figures 2E, 2F, 2G, 2H). Two types of hairy assemblies were found in the microscopic observation of leaves, one similar to flagpole-shaped head (Figures 3A, 3B) and another bamboo shoot shaped (Figures 3C, 3D), the latter is mostly deposited in the blade surface in the SH41.

### PCR identification and DNA blot analysis of ATM transgenic *Salvia*

**Figure 4:**
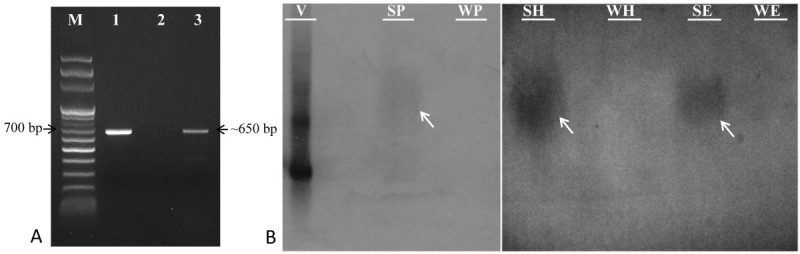
**Molecular analysis of ATM transformed** ***S. miltiorrhiza*** **. (A)** PCR analysis of transformerd *Salvia* plant. Genomic DNA was amplified using *hpt* II gene specific primers. Approx. 650 bp amplification was observed in transformed plant. Lanes: (M) Molecular weight marker, (1) Positive control (pTAG8 plasmid), (2) Wild type *Salvia*; and (3) Active mutagenic *Salvia* plant (SH41). **(B)** Confirmation of activation-tagged *S. miltiorrhiza* Bunge by Southern blot analysis. Genomic DNA was extracted from leaves and *hpt* II gene used as a probe. Hybridization was performed at 52°C. V- pTAG-8; SP- SH41 and WP- WT digested by *Pst* I; SH- SH41 and WH- WT digested by *Hind* III; SE- SH41 and WE- WT digested by *EcoR* I. Arrows indicate hybridization signals.

PCR analysis with *hpt* II gene specific primers was carried out to confirm the presence of the activation tagging T-DNA insertion in the genome of the putative transformant. The 650 bp expected *hpt* II fragment was found in the positive control (pTAG8 plasmid) as well as in transformed *Salvia* SH41. No amplification was observed in wild type plant (control) (Figure 4A).

Southern hybridization analysis was performed in order to estimate the number of inserted loci. Figure 4B showed the Southern hybridization pattern obtained with *hpt* II probe. The result showed single insertion in SH41 and no band was observed in the non-transformed control plant (Figure 4B).

### Compound extraction and HPLC analysis

For optimal extraction of active constituents from the *Salvia* plant, different solvent system was tested. The addition of formic acid improved the extraction efficiency of salvianolic acid B and Danshensu. As the result, 60% ethanol with 0.05% formic acid was the best solvent system (Table 1). The tanshinone I and tanshinone IIA detected in SH41 was 3.7 and 2 fold higher than wild type plant, respectively. These compounds were also greater than CK (2 fold and 3 fold higher, respectively). Salvianolic acid B was also found 1.4 and 2.4 fold higher in SH41 as compared to wild plant and CK, respectively. Other compounds like Danshensu, rosmarinic acid and salvianolic acid A were slightly higher in SH41, however, salvianolic acid A was less in SH41 as compared to CK. On the other hand, protocatechuic, protocatechuic aldehyde and caffeic acid were not detected in SH41 (Table 2).

**Table 1:** **Different solvent extractions of** ***Salvia miltiorrhiza*** **root (commercially available plant)**

Compounds	100% CH	70% CH	70% CH/F	100% Et	60% Et	60% Et/F
**Danshensu**	0.63 ± 0.06	1.05 ± 0.12	1.22 ± 0.02	0.36 ± 0.04	0.98 ± 0.04	1.30 ± 0.04
**Protocatechuic**	0.10 ± 0.01	0.13 ± 0.01	0.12 ± 0.01	0.09 ± 0.03	0.13 ± 0.13	0.14 ± 0.14
**Protocatechuic aldehyde**	0.06 ± 0.05	0.06 ± 0.01	0.06 ± 0.03	0.06 ± 0.02	0.06 ± 0.06	0.06 ± 0.06
**Caffeic acid**	0.06 ± 0.05	0.06 ± 0.02	0.06 ± 0.03	0.05 ± 0.02	0.06 ± 0.06	0.06 ± 0.06
**Rosmarinic acid**	0.65 ± 0.07	1.08 ± 0.08	1.20 ± 0.02	0.36 ± 0.03	0.96 ± 0.02	1.21 ± 0.04
**Salvianolic acid B**	3.35 ± 0.09	5.43 ± 0.04	5.97 ± 0.08	1.68 ± 0.08	4.87 ± 0.07	6.08 ± 0.06
**Salvianolic acid A**	0.25 ± 0.03	0.36 ± 0.04	0.39 ± 0.04	0.16 ± 0.01	0.33 ± 0.03	0.41 ± 0.02
**Cryptotanshinone**	0.03 ± 0.01	0.06 ± 0.02	0.07 ± 0.02	0.07 ± 0.03	0.06 ± 0.01	0.07 ± 0.02
**Tanshinone I**	0.08 ± 0.03	0.10 ± 0.04	0.11 ± 0.03	0.18 ± 0.04	0.07 ± 0.02	0.14 ± 0.05
**Tanshinone IIA**	0.04 ± 0.01	0.07 ± 0.02	0.08 ± 0.02	0.15 ± 0.04	0.07 ± 0.01	0.14 ± 0.03

*CH*, Methanol; *CH/F*, Methanol with 0.05% formic acid; *Et*, Ethanol; *Et/F*, Ethanol with 0.05% formic acid. Data expressed as mean ± SD for 3 repeats (mg/g).

**Table 2:** **HPLC-UV detection of** ***Salvia*** **roots extracted with 60% ethanol containing 0.05% formic acid for seven water-soluble and three fat-soluble ingredients, (** ***n*** **= 3) (mean ± SD; mg/g extract)**

Constituents	WTR	SHR	CK
**Danshensu**	0.19 ± 0.07	0.28 ± 0.08	1.30 ± 0.17
**Protocatechuic**	ND	ND	0.14 ± 0.04
**Protocatechuic aldehyde**	0.08 ± 0.01	ND	0.06 ± 0.01
**Caffeic acid**	ND	ND	0.06 ± 0.04
**Rosmarinic acid**	2.11 ± 0.21	2.87 ± 0.74	1.21 ± 0.47
**Salvianolic acid B**	10.44 ± 0.75	14.45 ± 0.37	6.08 ± 0.39
**Salvianolic acid A**	0.12 ± 0.07	0.16 ± 0.09	0.41 ± 0.04
**Cryptotanshinone**	0.59 ± 0.13	0.34 ± 0.08	0.07 ± 0.05
**Tanshinone I**	0.08 ± 0.01	0.30 ± 0.05	0.14 ± 0.06
**Tanshinone IIA**	0.20 ± 0.09	0.42 ± 0.09	0.14 ± 0.04

*WTR* Wild type root, *SHR* SH41 root, *CK* Commercially available *Salvia miltiorrhiza* root, *ND* Non detectable.

## Discussion

A new biotechnological method of mutagenesis, called activation tagging mutagenesis (ATM), has increased the understanding of development in various plants by intensifying the range of gene expressions and accumulation of secondary metabolites. This technique exploits the transferred DNA (T-DNA) from *Agrobacterium tumefaciens* to introduce a viral CaMV35S enhancer region randomly into the genome. The enhancer may dominantly “activate” and/or widen the pattern of expression of a gene that is near the enhancer of the introduced T-DNA (Hsing et al. 2007). It has been reported that the CaMV35S enhancer activate the expression of genes located upstream or downstream of T-DNA in *Arabidopsis* and rice (Ichikawa et al. 2003; Jeong et al. 2006). The insertion of an enhancer sequence in the vicinity of an endogenous gene can alter the transcriptional pattern of the gene, resulting in a mutant phenotype. Activation tagging has been undertaken extensively in a number of dicot and monocot plants (Michael and Anthony 2011).

*Salvia miltiorrhiza* (Danshen) is an important Chinese medicinal herb and diterpene tanshinones are its major active compounds. In previous studies, *in vitro* methods such as hairy root cultures (Hu et al. 1993; Ge and Wu 2005a; Yan et al. 2005), cell suspension cultures (Chen et al. 1997), addition of elicitors and optimization of media (Wu et al. 2003; Zhang et al. 2004; Ge and Wu 2005b; Gupta et al. 2011), have been reported. In our previous study, ATM transgenic callus lines of *Salvia* with greater tanshinones content were developed (Lee et al. 2008). In the present study, one of the 4 months old ATM transgenic (SH41) with an altered phenotype was identified and subjected to quantitative analysis of medicinally important ingredients. Insertion of T-DNA was confirmed by PCR with hygromycin phosphotransferase (*hpt* II ) gene specific primers and Southern blot analysis. PCR analysis showed amplification of *hpt* II gene which confirmed the presence of an activation tagging T-DNA in SH41. DNA blot analysis showed single copy insertion of transgene in SH41. Single or low-copy integration of transgenes through *Agrobacterium* have been observed in several plant species (Find et al. 2005; Maghuly et al. 2006; Tsay et al. 2012). Microscopic (SEM) analysis showed adaxial and abaxial surface of transgenic *Salvia* leaves has more trichomes as well as bigger and wobbly guard cells and stomata. In contrast, wild type plant showed the less number of trichomes and smaller guard cells and stomata. Genetic modification in plants leads to change in morphology. Ectopic expression of gene in *Arabidopsis* showed different morphology and abnormal flowers containing wrinkled organs (Liu et al. 2008). Weigel et al. (2000) reported that the 35S enhancer element might increase the expression of nearby genes without altering the original expression pattern. Since, incorporation of CaMV35S enhancers of pTAG8 is random in the genome, so changes in leaf morphology of SH41 is may be due to the activation of genes responsible for leaf development, which remains to be confirmed.

Based on previous studies, phenolic acids and diterpene contents vary with different solvent systems and extraction methods. In the present study, the combinations of methanol and ethanol with water were used to recover greater yield of both water as well as lipid soluble constituents of *Salvia* roots by sonication. It was observed that, 60% ethanol containing 0.05% formic acid was the best extraction system and the addition of formic acid increased the extraction efficiency (Table 1). Although, the effect of temperature on extraction were reported in other studies, such as highest content of salvianolic acid B (48.37 ± 1.21 mg/g) was yielded in microwave assisted extraction with water (MAE-W) at 75°C, or the highest contents of tanshinones including dihydrotanshinone, cryptotanshinone, tanshinone I, and tanshinone IIA were obtained in MAE-W at 100°C (Zhou et al. 2012), but we needed a method to extract salvianolic acid B and tanshinones simultaneously. Therefore, 60% ethanol containing 0.05% formic acid was the best choice for this study.

The active compound of *S. miltiorrhiza*, tanshinone, was proved with lots of biological activities. For instance, tanshinone I could enhance learning and memory (Kim et al. 2009), while tanshinone IIA has anti-oxidative (Wang et al. 2003; Yang et al. 2008), anti-inflammatory (Jang et al. 2006), anti-proliferative (Liu et al. 2006) and anti-tumor properties (Dong et al. 2007). Therefore, *S. miltiorrhiza* has been widely used to produce a number of traditional Chinese medicine preparations. However, because of low content, the supplement of tanshinone for the increasing needs of clinical applications has become a research attention. Therefore, development of transgenic *Salvia* with altered tanshinone content could overcome the shortage of traditional Chinese medicine preparations. Manipulating multiple genes at multiple control sites in desired metabolite production may cause metabolic flux alteration (Nims et al. 2006). The activation tagging has the ability to activate whole branches of biochemical pathways leading to the accumulation of several subclasses of natural product usually present in low concentration (Tani et al. 2004). In this study ATM transgenic of *Salvia* showed significant increase in diterpene tanshinone contents in roots as compared to wild plant. Tanshinone I, tanshinone IIA and salvianolic acid B were 3.7, 2 and 1.4 fold higher in SH41 as compared to wild plant. In other study it was reported that the over-expression of genes encoding 3-hydroxy-3-methylglutaryl CoA reductase (*SmHMGR*), 1-deoxy-D-xylulose-5-phosphatesynthase (*SmDXS*) and geranylgeranyl diphosphate synthase (*SmGGPPS*) in *S. miltiorrhiza* can significantly enhance the production of tanshinones in roots to levels higher than that of the control. *S. miltiorrhiza* plants over-expressing *SmHMGR*, *SmGGPS* and *SmDXS* produced 2.1 to 5.7 fold higher tanshinones than control plants (Kai et al. 2011).

## Conclusions

In conclusion, the current study provides good basement and helpful information for commercial large-scale production of tanshinones in the roots of *Salvia* by developing ATM transgenic lines with altered phenotype. Also, the extraction procedure and quantitative HPLC procedures were developed for determining liposoluble as well as water-soluble phenolic acid compounds.

## Authors’ original submitted files for images

Below are the links to the authors’ original submitted files for images. Authors’ original file for figure 1 Authors’ original file for figure 2 Authors’ original file for figure 3 Authors’ original file for figure 4 Authors’ original file for figure 5 Authors’ original file for figure 6

## Abbreviations

ATM: Activation tagging mutagenesis
HPLC: High performance liquid chromatography
hptII: Hygromycin phosphotransferase II gene
SEM: Scanning electron microscopy.
