# Supplementary figures and images for: Activation tagging in Salvia miltiorrhiza can cause increased leaf size and accumulation of tanshinone I and IIA in its roots
Source: Bot Stud. 2013 Sep 24;54:37. doi: 10.1186/1999-3110-54-37 (PMC5432761; doi:10.1186/1999-3110-54-37)

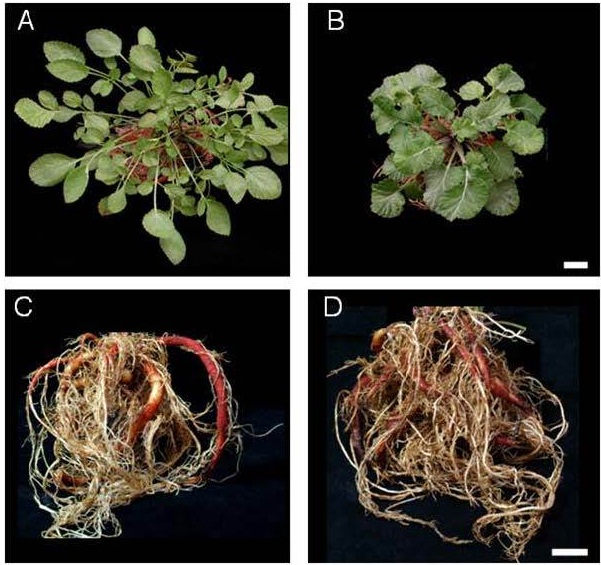

Supplement: Supplementary file 1 — Authors’ original file for figure 1 [file 40529_2013_33_MOESM1_ESM.jpeg]

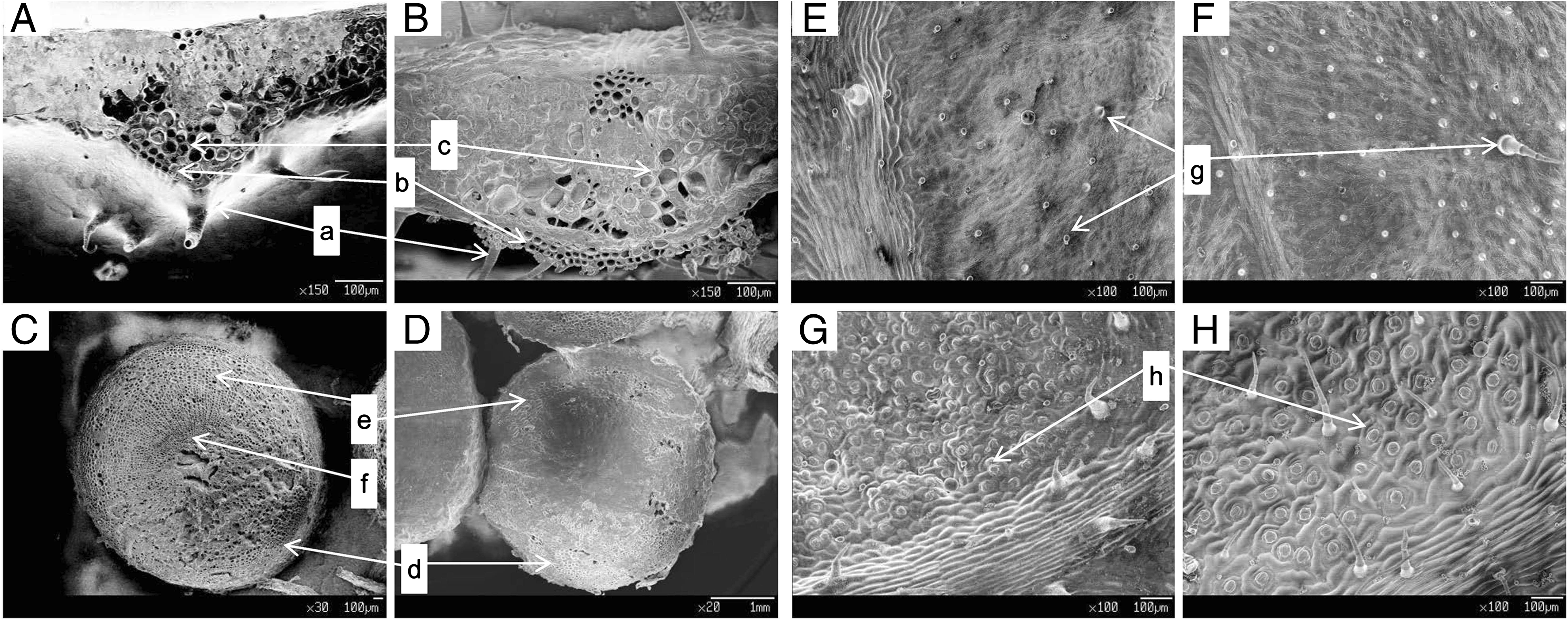

Supplement: Supplementary file 2 — Authors’ original file for figure 2 [file 40529_2013_33_MOESM2_ESM.tiff]

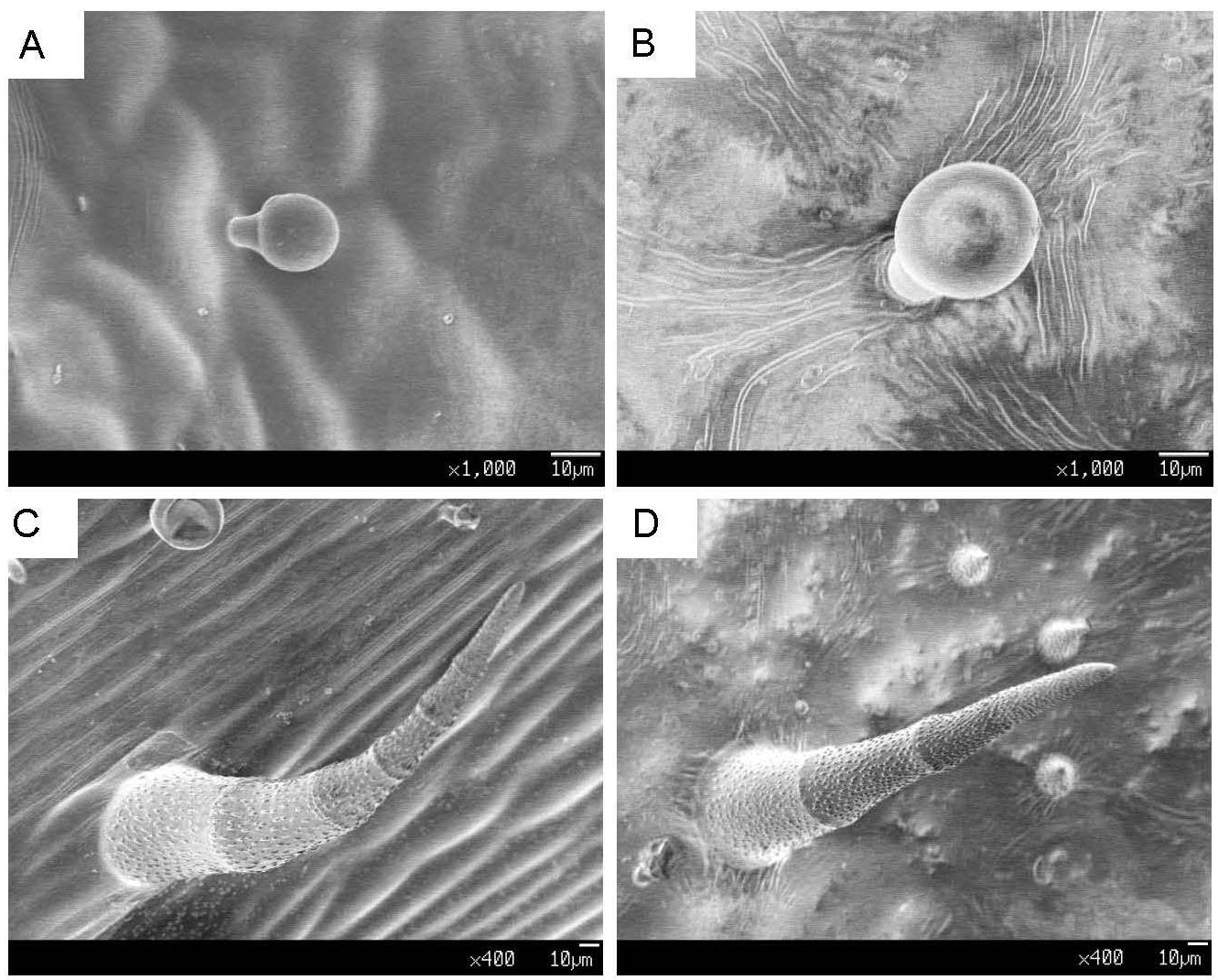

Supplement: Supplementary file 3 — Authors’ original file for figure 3 [file 40529_2013_33_MOESM3_ESM.jpeg]

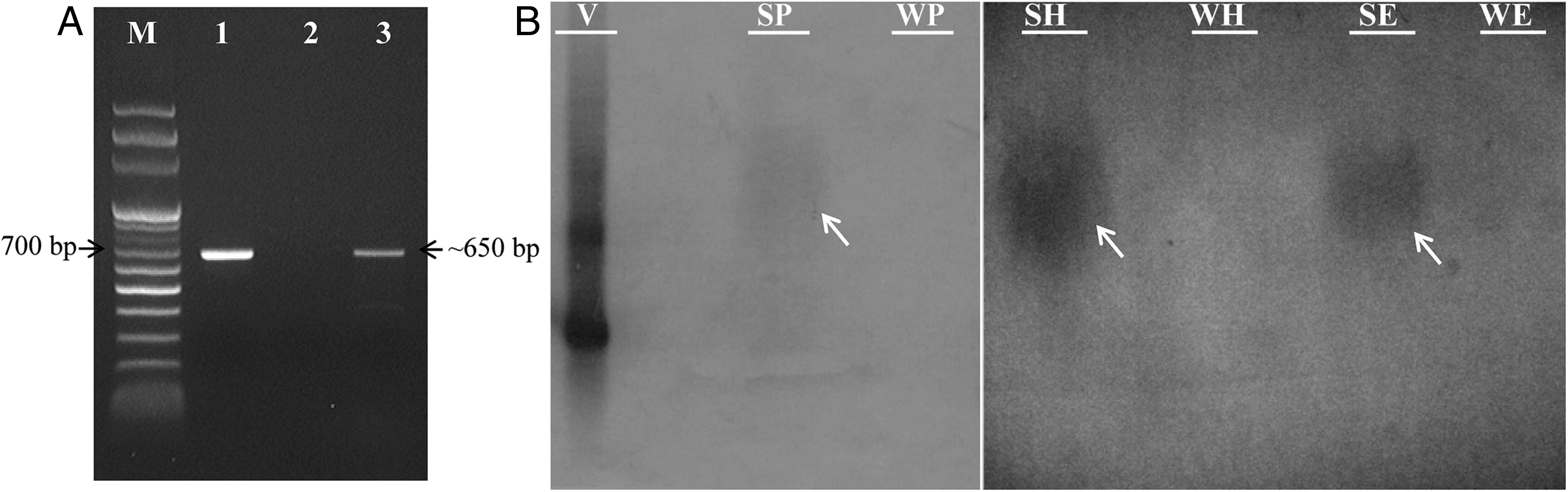

Supplement: Supplementary file 4 — Authors’ original file for figure 4 [file 40529_2013_33_MOESM4_ESM.tiff]

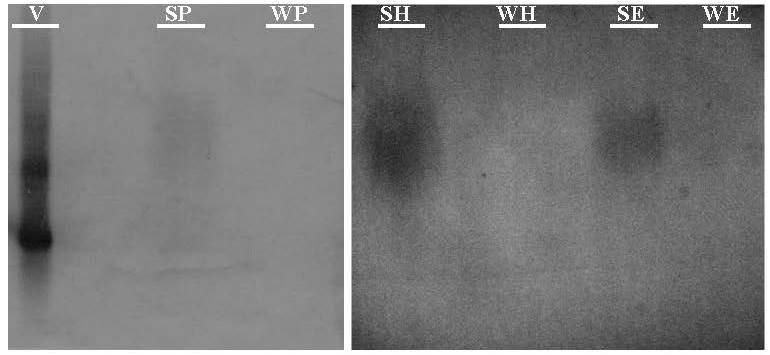

Supplement: Supplementary file 5 — Authors’ original file for figure 5 [file 40529_2013_33_MOESM5_ESM.jpeg]

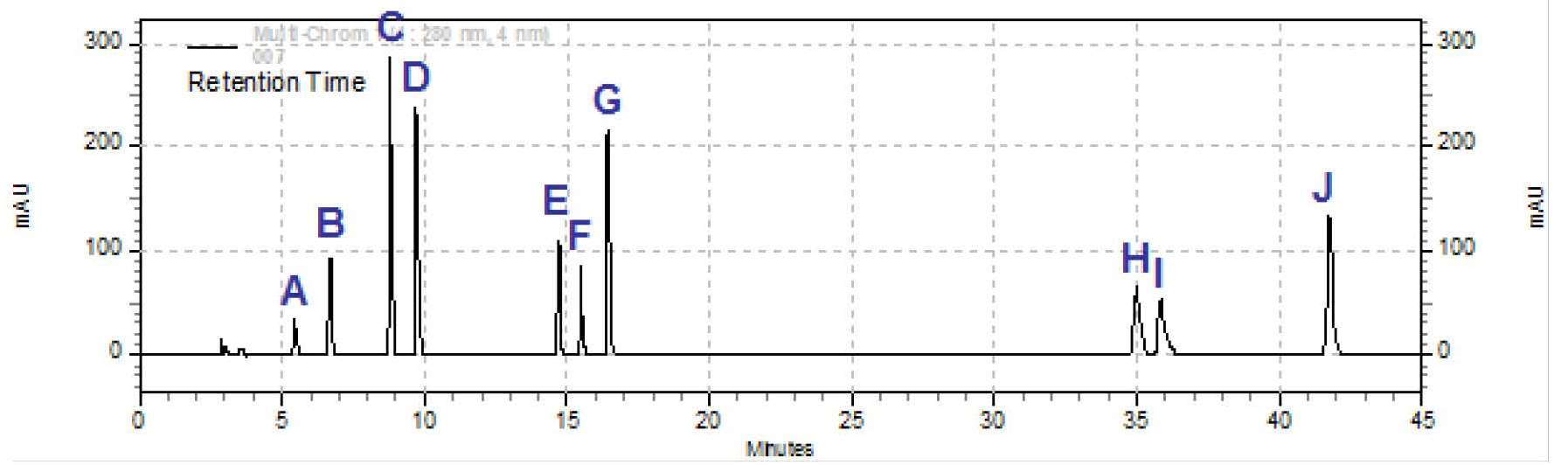

Supplement: Supplementary file 6 — Authors’ original file for figure 6 [file 40529_2013_33_MOESM6_ESM.jpeg]
